# Effect of a Dual Functional Polymer on the Electro-Optical Properties of Blue Phase Liquid Crystals

**DOI:** 10.3390/polym11071128

**Published:** 2019-07-03

**Authors:** Liang Gao, Ke-Meng Wang, Rui Zhao, Hong-Mei Ma, Yu-Bao Sun

**Affiliations:** 1 School of Electronic and Information Engineering, Hebei University of Technology, Tianjin 300401, China; 2 Department of Applied Physics, Hebei University of Technology, Tianjin 300401, China

**Keywords:** blue phase, ethynyl group, polymer system, Kerr effect, liquid crystal devices

## Abstract

The effects of the fluorinated monomer with an ethynyl group on the electro-optical properties of polymer-stabilized blue phase liquid crystals (PSBPLCs) were investigated in different polymer systems. In rigid polymer systems, the Kerr constant can be increased by about 27.6%, while keeping a microsecond response time. In soft polymer systems, hysteresis decreased by about 45.5% and residual birefringence can be reduced from 1.85% to 0.6%. The above phenomena exhibited dual functions of affecting the anchoring energy and the viscosity of the system simultaneously. The results provide a potential value for the ethynyl-containing monomers in PSBPLC systems.

## 1. Introduction

Blue phase liquid crystal (BPLC) is a particular frustrated phase that exists in a narrow temperature range between the isotropic phase (ISO) and chiral nematic phase (N*) [1,2,3,4,5]. BPLCs can be classified into the following three types with the increased temperature: BP I, BP II, and BP III [6,7,8,9]. Among them, BP I and BP II are body-centered cubic structures and simple cubic structures, respectively, and BP III has an amorphous structure with the same symmetry as the isotropic phase [3,4,10,11,12]. BPLCs have attracted much attention because of their nano-scale periodic lattice structure and unique features, including optically isotropic, microsecond response speed, and stimuli-response Bragg reflection [13,14,15,16]. Among these features, the fast response is particularly attractive, because it enables field-sequential color displays with negligible color breakup [17,18,19]. However, the narrower temperature range of BPLCs hinders its utility in practice [1,4,5]. To tackle this problem, the polymer stabilization (PS) BPLCs were proposed to broaden the temperature range over 60 K, including room temperature [1]. Based on this method, PSBPLCs open a pathway for advanced LC displays [18,20] and tunable photonic devices [21,22,23].

However, some drawbacks need to be solved, based on this method, such as high operating voltage, noticeable hysteresis, relatively low transmittance, and long-term stability, before PSBPLC devices can be widely used [2,4,11,19]. The key issue here is the operating voltage [6]. The operating voltage needs to be reduced to be less than 10 V in order to fit the conventional amorphous thin-film transistor, and the operating electric field needs to be less than the critical field to eliminate the hysteresis effect [24,25,26]. A lot of approaches have been proposed, from the design of the device to the synthesis of novel material structures [27,28,29,30]. The reduced operating voltage can be achieved by fabricating the protrusion electrodes or applying a vertical field switching mode for increasing the effective electric field of the LC layer, and using large Kerr constant (K) BPLCs for enhancing the Kerr effect of the system.

Generally, the operating voltage is inversely proportional to the K^−1/2^ of PSBPLCs [27]. That is to say, increasing the Kerr constant helps to reduce the operating voltage. Gerber found that the Kerr constant can be expressed as follows [7]:

(1) $$K∼\Delta n*\Delta \varepsilon \frac{\varepsilon_{0}P^{2}}{k\lambda {\left(2\pi \right)}^{2}},$$

where ∆*n*, ∆*ε,* and *k* are the birefringence, dielectric anisotropy, and average elastic constant of the host LC, respectively, and *P* is the pitch. Increasing the ∆*n* and ∆*ε* by introducing polar groups is the most effective way to increase the Kerr constant. However, the disadvantage is the increased viscosity (*γ*_1_) due to the presence of several polar groups [29]. The relaxation time (*τ*) of PSBPLCs is related to the LC parameters of *γ*_1_, *k*, and *P* [6,17], as follows:

(2) $$\tau =\frac{\gamma_{1}P^{2}}{k{\left(2\pi \right)}^{2}},$$

Combining Equations (1) and (2) indicates that the realization of a low operating voltage and fast response needs to balance various factors in PSBPLCs. Therefore, it is imperative to investigate the influence of the polymer system on the electro-optical (EO) properties of PSBPLCs, especially the role of the doping system in polymer systems. However, the underlying physical mechanisms of monomers with an ethynyl group in PSBPLCs are rarely reported.

In this article, we investigated the influences of the fluorinated ethynyl-containing monomer, 1-Ethynyl-4-(trifluoromethyl)benzene (ETB), on the EO properties of PSBPLCs. For the rigid polymer systems, the operating voltage was reduced, while the response time was exhibited in the microsecond range. For the soft polymer systems, the doping monomer favored the reduced hysteresis and residual birefringence, but the tradeoff was the increased response time. This implies that ETB performs dual functions in different polymer systems, which affects the interaction between polymer system and LCs after the polymerization process, thus exhibiting different response characteristics. 

## 2. Materials and Methods

**Figure 1:**
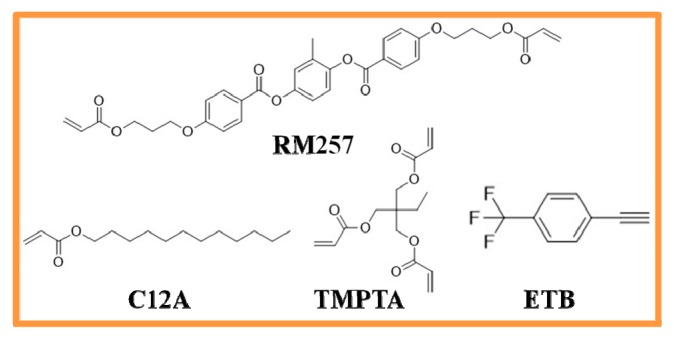
The chemical structures of polymer used in this work. ETB—1-Ethynyl-4-(trifluoromethyl)benzene. TMPTA—1,1,1-Trimethylolpropane Triacrylate.

In order to study the role of ETB in different polymer systems, rigid and soft polymer systems were prepared by incorporating ETB, respectively. The BPLC used in this work was composed of 96.3 wt % nematic liquid crystal host TEB300 (∆*ε* = 29.3, ∆*n*= 0.166, Slichem Co., Ltd., Shijiazhuang, China) and 3.7 wt % chiral dopant R5011 (HCCH, Nanjing, China). To form PSBPLCs, four kinds of polymers with different functional groups—a di-functional monomer RM257, ultraviolet (UV) curable monomers C12A, TMPTA (1,1,1-Trimethylolpropane Triacrylate, HCCH), and ETB (1-Ethynyl-4-(trifluoromethyl)benzene, AnHui JiDa Pharm&Chem Co., Ltd., Shanghai, China)—were used to stabilize the disclinations of BPLCs. The chemical structures are illustrated in Figure 1. The increase in functional groups and decrease in chain length of monomers contribute to forming a tougher polymer network. Therefore, the above materials constitute two different kinds of polymer systems, namely: rigid polymer (RP) systems (RM257/TMPTA/ETB) and soft polymer (SP) systems (RM257/C12A/ETB). To promote the UV polymerization rate, a small amount of IRG184 was added in all of the samples. For comparison, the total concentration of the polymer was kept in the same condition, as listed in Table 1.

**Figure 2:**
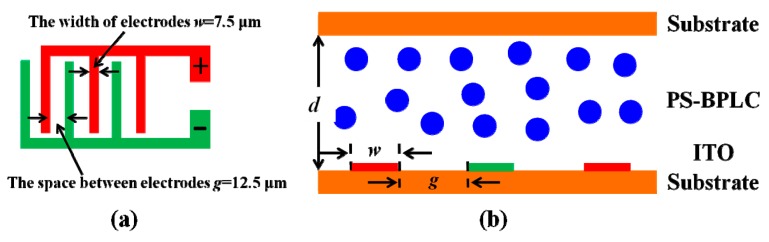
The IPS cells of (**a**) device configurations and (**b**) the cross-sectional view without electric fields. PSBPLC—polymer-stabilized blue phase liquid crystals.

After the homogeneous mixing, the precursor was injected into an in-plane switching (IPS) cell without special surface treatment. The cell had an electrode width of w = 7.5 μm, electrode gap of g = 12.5 μm, and cell gap of d = 7.5 μm, as shown in Figure 2. The samples were all placed in the temperature controller at the cooling rate of 0.3 K/min. Then, a 365 nm UV lamp was used to cure the samples with 8 mW/cm^2^ for 30 min, before the BPLC was transferred to the chiral nematic phase (N*). After the UV process, the nanostructured PSBPLCs were formed. The excellent blue phase textures of these samples were observed before and after UV process. It illustrated that BPLCs can be well stabilized by the polymer systems. Furthermore, all of the samples exhibit a wide temperature range of more than 60 K, including room temperature after the UV process. To test the electro-optical properties, the samples were sandwiched between a crossed polarizer, and the direction of the electrodes was set at 45° from the transmission axis of the polarizer. The IPS cell was applied with a 1 kHz square-wave voltage after amplifying the signal. A halogen lamp was used as the probing light, and the transmitted light signal was collected by a photodiode connected to a spectrometer. To investigate the hysteresis, the voltage-dependent transmittance (VT) curves in the forward and backward scans were recorded.

## 3. Results

### 3.1. Kerr Effect

**Figure 3:**
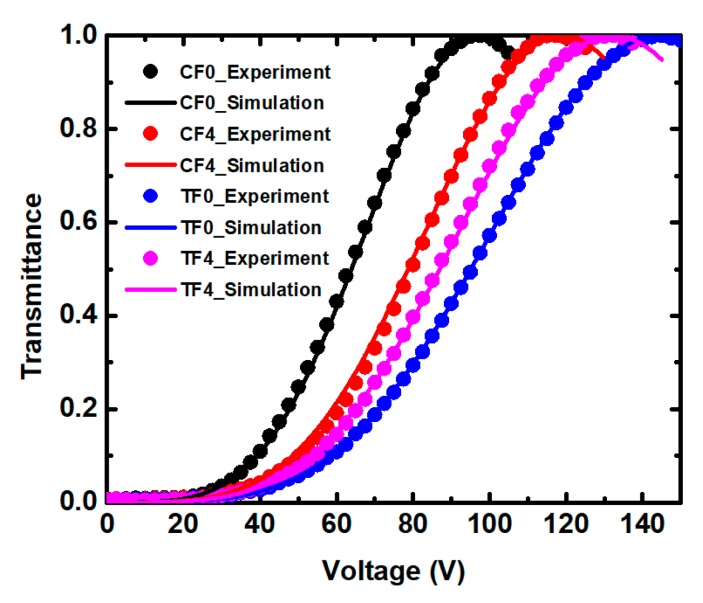
The normalized voltage-dependent transmittance (VT) curves for four samples at room temperature (*λ* = 632.1 nm).

**Figure 4:**
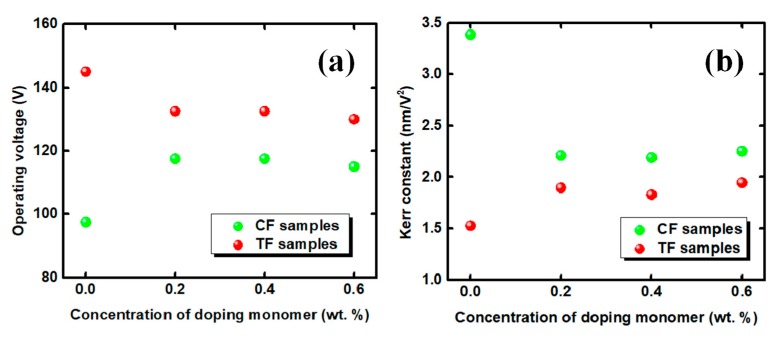
All of the samples of (**a**) the operating voltages and (**b**) Kerr constants in different polymer systems.

Figure 3 compares the normalized VT curves of four samples in the RP systems and SP systems. Because of the limitation of space, we only depicted four samples in Figure 3. The operating voltages at the maximum transmittance of the ETB-containing samples showed a smaller value compared with the undoped sample (TF0). In the TF samples, the voltage was reduced from 145 V for the undoped sample (TF0) to 130 V for the sample (TF6) doped with 0.6% ETB, as shown in Figure 4a. The operating voltage was reduced by about 10.3% compared with the undoped sample (TF0). In contrast, the operating voltage increased from 97.5 V for the sample CF0 to 117.5 V for the sample CF4, with the additional of ETB in the CF samples. This illustrates that the electro-optical properties exhibited a significant difference in the RP and SP systems. In addition, a slight voltage drop trend was also observed when the ETB increased from 0.2 to 0.6 wt %. This result implied that the fluorinated ETB also played an important role in reducing the operating voltage, because of its lubrication effect [30].

The induced birefringence (∆*n_ind_*) of the BPLCs can be expressed as the extended Kerr model [20,31], as follows:

(3) $$\Delta n_{ind}=\Delta n_{s}\left(1-\exp\left[-{\left(\frac{E}{E_{s}}\right)}^{2}\right]\right),$$

where ∆*n_s_* represents the saturated induced birefringence, *E* stands for the applied electric field, and *E_s_* is the saturation field. As the electric field increases, the induced birefringence ∆*n_ind_* gradually tends to the saturated birefringence ∆*n_s_*. Under the weak electric field, the Kerr constant can be obtained as follows [6]:

(4) K=Δns(λEs2).

In Figure 3, the solid lines represent the fitting curves based on the extended Kerr model. The simulated results could be obtained to fit the experiments using the commercial software (Techwiz LCD 3D, Sanayi System, Incheon, Korea) by adjusting ∆*n_s_* and *E_s_*. As shown in Figure 4b, the Kerr constants of the TF samples were smaller than that of the CF sample, which is attributed to the higher surface anchoring energy of the TF samples. However, the effect of ETB on the Kerr constant was opposite to the other polymer systems. The Kerr constant of TF0 was smaller than that of the samples in the RP systems, because the tri-functional monomer TMPTA-formed polymer network was tougher than the mono-functional monomer ETB. Although both C12A and ETB were mono-functional monomers, the benzene ring-containing ETB exhibited a stronger anchoring energy than C12A, with flexible chains in the SP systems, resulting in a decrease in the Kerr constant. 

### 3.2. Hysteresis

**Figure 5:**
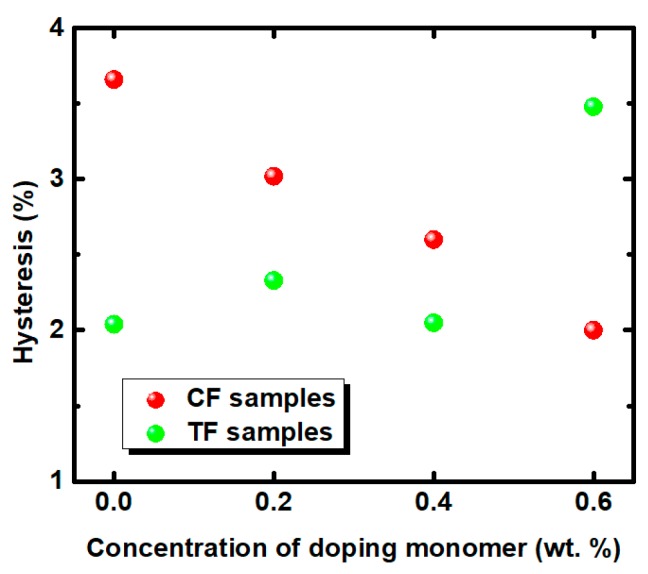
The resulting hysteresis for all samples in the different polymer systems.

**Figure 6:**
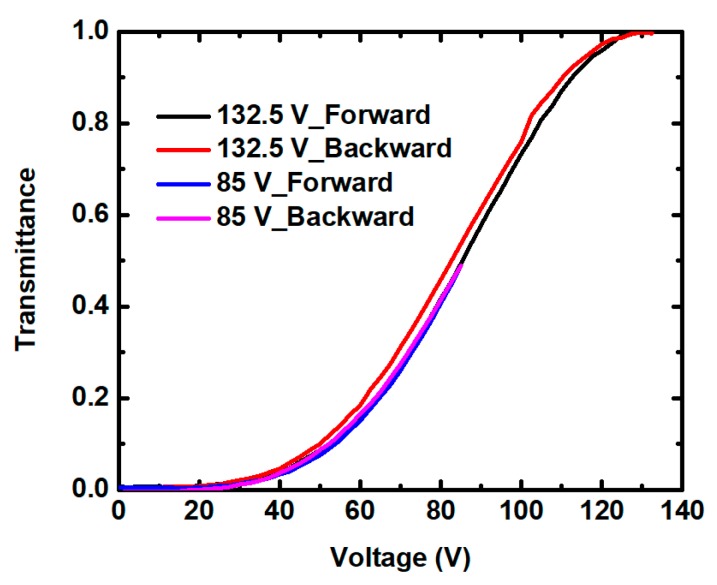
Measured hysteresis of sample TF4 with different applied voltages.

The hysteresis-free device is in urgent need in order to improve the gray-scale response. Hysteresis is defined as ∆*V*/*V_on_*, where ∆*V* is the voltage difference between the upward and backward loops at 50% peak transmittance, and *V_on_* is the operating voltage. Figure 5 depicts the hysteresis results of all of the samples. Obviously, the effect of ETB was not only different in voltage, but also in hysteresis for the RP and SP systems. Different hysteresis variations are observed in both SP and RP systems, with the increased ETB content. This result may be due to the dual functions of ETB on polymer network and BPLCs simultaneously [26]. For the TF samples, the hysteresis effects of the samples of TF2, TF4, and TF6 were more obvious compared with the TF0 without ETB. However, the resulting hysteresis can be suppressed in the CF samples. In particular, the hysteresis of sample CF6 decreased by 45.4% compared with that of sample CF0 without ETB. In addition, with the consideration of the correlation between the peak electric field and hysteresis, the hysteresis of sample TF4 was measured with different applied voltages. In Figure 6, sample TF4 showed an obvious hysteresis loop for a full transmittance cycle. However, the forward and backward curves fit well, and a hysteresis-free device was obtained when the operating voltage was decreased to 85 V.

### 3.3. Residual Birefringence

**Figure 7:**
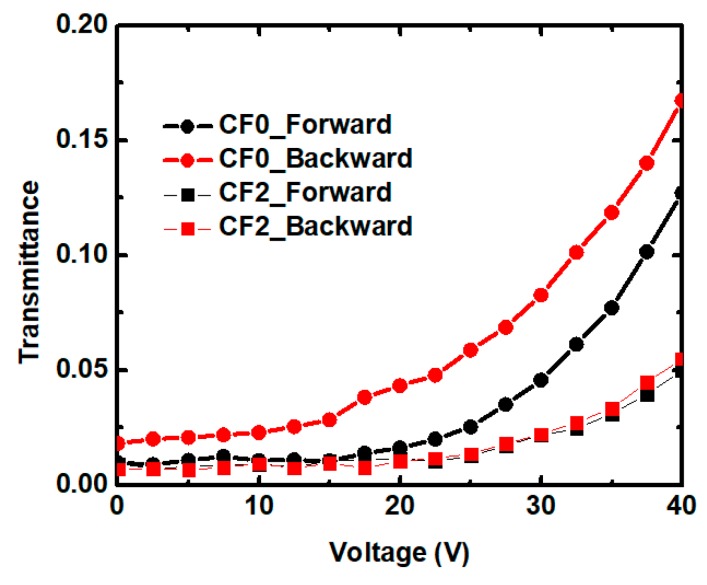
The residual birefringence of samples CF0 and CF2, respectively.

Residual birefringence is a serious issue that degrades the contrast ratio, and it should be addressed in PSBPLC devices. Residual birefringence is defined as transmittance backward at 0 V. According to the measured VT curves, sample CF0 had the largest residual birefringence in the CF samples, and can be reduced from 1.85% to 0.6% with the increasing ETB, as shown in Figure 7. However, ETB had almost no remarkable influence on the residual birefringence of the TF samples. So, the interaction between the liquid crystals and polymers had an important effect on residual birefringence. The residual birefringence of the TF samples (or RP systems) can be maintained because of the stiffness of the polymer network; on the contrary, the CF samples (or SP systems) are not tough enough to recover the initial state once the electric field exceeds a critical field. It indicates that the mixed ETB in SP systems helps to suppress the light leakage at the dark state and reduce the residual birefringence.

### 3.4. Response Time

**Figure 8:**
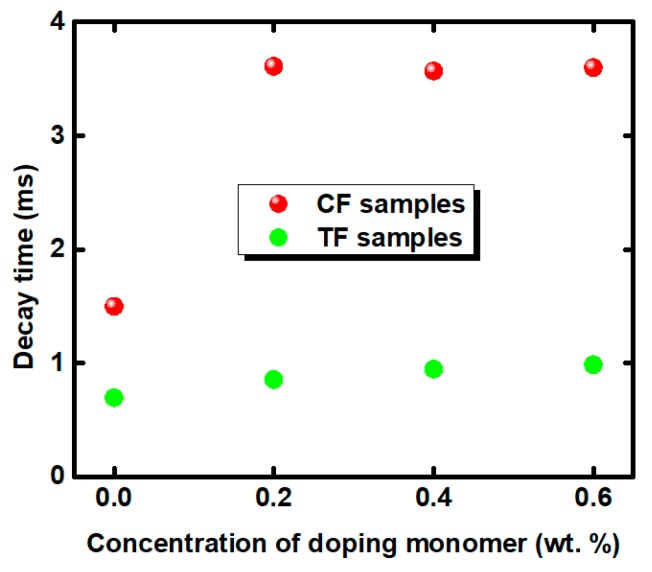
Electro-optical response of the samples in different polymer systems.

Fast response is another revolutionary feature for the next-generation LC displays, because it enables color-sequential display while eliminating color filters. Decay time is defined as the transmittance from 90% to 10%. As shown in Figure 8, the decay time of the CF samples were slower than the TF samples, because of the lower anchoring energy in the SP systems. Sample TF0 had the fastest response speed because of the strongest polymer network formed by RM257 and TMPTA. However, the decay time of all of the samples was increased with the introduction of ETB. Although we have previously shown that ETB enhanced the surface anchoring energy of the SF system, it was not afforded to decrease the response time. This implies that ETB is not involved totally during the UV process, because the required energy is higher for the ethynyl groups when the polymerization occurs. The presence of additional ETB may increase the viscosity of the system, resulting in an increased response time based on the Equation (2).

**Figure 9:**
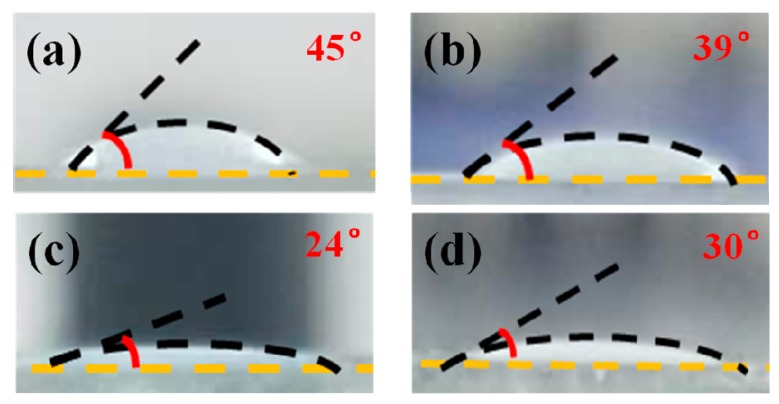
Contact angle of the LC host/polymer film surfaces, formed by (**a**) C12A, (**b**) C12A + 10 wt % ETB, (**c**) TMPTA, and (**d**) TMPTA + 10 wt % ETB. The corresponding contact angles are labeled at the top right corner of the photos.

**Figure 10:**
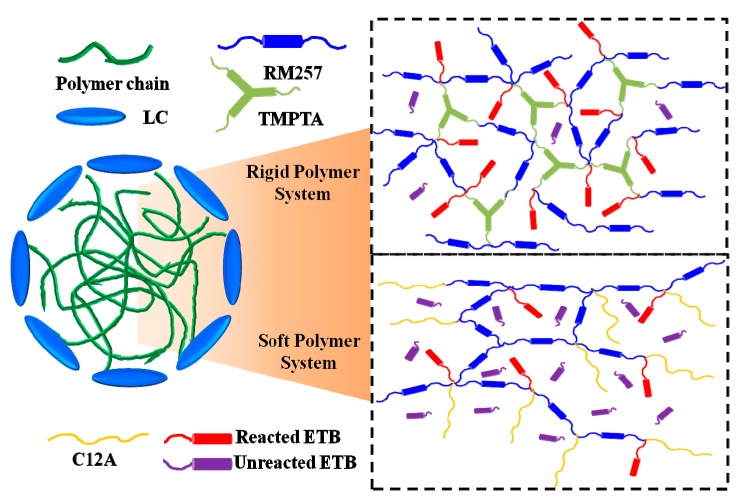
Schematic diagrams of the mechanism of ETB in different polymer systems.

The effect of ETB on the anchoring energy can be determined by the contact angle of the LC host on the different polymer films, as shown in Figure 9. The contact angle of the LC host decreased from 45° to 39° on the polymer film formed by SP systems, but increased from 24° to 30° on the polymer film formed by the RP systems. The results indicate that the anchoring energy becomes stronger with the increased ETB in the SP systems. However, the effect of ETB is reversed in the RP systems. To understand the change in EO properties for different polymer systems, schematic diagrams of the mechanism of ETB were proposed, as depicted in Figure 10. The reactive mesogen RM257 was used to form the 3D polymer network. Two different monomers, C12A and TMPTA, were used to stabilize the BPLCs to form RP systems and SP systems, respectively. For investigating the effect of ETB on different polymer systems, ETB was added in the PSBPLCs. The EO properties showed that ETB has dual functions of affecting the anchoring energy in different polymer networks, and increasing the viscosity caused by incomplete polymerization. On the one hand, ETB provides a stronger anchoring energy in SP systems, but it holds the opposite effect on the RP systems, as shown in Figure 9. On the other hand, the increased response time indicates that the viscosity is increased because of the unreacted ETB in the PSBPLCs. In addition, the response time of the SP systems changed dramatically compared with that of the RP systems. This may be because ETB is favoured in RP systems, and the dense polymer network helps the existence of the ETB formed by tri-functional TMPTA. 

## 4. Conclusions

In conclusion, we reported the effect of the fluorinated monomer with an ethynyl group on the electro-optical properties of PSBPLC in different polymer systems. The Kerr effect can be enhanced and the operating voltage can be decreased by about 11.5%, while keeping a microsecond response in the RP systems. For the SP systems, both hysteresis and residual birefringence can be reduced, while the tradeoff is the increased operating voltage. The response time of all of the samples increased with the addition of ETB in different systems. These results will contribute to the application of ethynyl-containing materials in PSBPLCs.

## Figures and Tables

**Table 1:** Compositions of polymer-stabilized blue phase liquid crystals (PSBPLCs) with two different kinds of polymer systems. ETB—1-Ethynyl-4-(trifluoromethyl)benzene. TMPTA—1,1,1-Trimethylolpropane Triacrylate.

Sample	BPLC (wt %)	RM257 (wt %)	C12A (wt %)	TMPTA (wt %)	ETB (wt %)	RG184 (wt %)
CF0	92.0	4.2	3.5	\	0	0.3
CF2			3.3	\	0.2	
CF4			3.1	\	0.4	
CF6			2.9	\	0.6	
TF0	92.0	4.2	\	3.5	0	0.3
TF2			\	3.3	0.2	
TF4			\	3.1	0.4	
TF6			\	2.9	0.6	
